# Analysis of the Clonality of *Candida tropicalis* Strains from a General Hospital in Beijing Using Multilocus Sequence Typing

**DOI:** 10.1371/journal.pone.0047767

**Published:** 2012-11-09

**Authors:** Yuan Wu, Haijian Zhou, Jing Wang, Lianqing Li, Wenge Li, Zhigang Cui, Xia Chen, Ruiqi Cen, Jinxing Lu, Ying Cheng

**Affiliations:** 1 State Key Laboratory for Infectious Disease Prevention and Control, National Institute for Communicable Disease Control and Prevention, Chinese Center for Disease Control and Prevention, Beijing, People's Republic of China; 2 China-Japan Friendship Hospital, Beijing, People's Republic of China; 3 Shanxi Center for Clinical Laboratories, TaiYuan, Shanxi, People's Republic of China; 4 Department of Critical Care Medicine, Fu Xing Hospital, Capital Medical University, Beijing, People's Republic of China; Duke University Medical Center, United States of America

## Abstract

Multilocus sequence typing (MLST) based on six loci was used to analyze the relationship of 58 *Candida tropicalis* isolates from individual patients in a general hospital in Beijing, China. A total of 52 diploid sequence types (DSTs) were generated by the MLST, all of which were new to the central database. Unweighted Pair Group Method with Arithmetic Mean (UPGMA) dendrograms were constructed, which showed that the 58 isolates were distributed robustly and 6 main groups were clustered regardless of the specimen source and medical department. The minimum spanning tree (MST) of the 58 isolates (52 DSTs) and all 401 isolates (268 DSTs) in the *C. tropicalis* central database (http://pubmlst.org/ctropicalis/) indicated that the isolates in this study clustered in three relative pure clonal complexes, and 2 clustered with isolates from Taiwan, Belgium, Brazil, and the US. This study presents the first MLST analysis of *C. tropicalis* isolates from Mainland China, which may be useful for further studies on the similarity, genetic relationship, and molecular epidemiology of *C. tropicalis* strains worldwide.

## Introduction

With the increasing number of immunocompromised patients, long-term hospitalized patients, and invasive medical inspection and therapy, the genus *Candida* has emerged as a major group of opportunistic pathogens that cause superficial and invasive infections in humans [1]–[5]. *Candida* is considered the fourth most commonly isolated organisms from nosocomial bloodstream infections in United States and sixth in Europe [6]–[10]. The invasive infections caused by *Candida* species are associated with significant morbidity and mortality [5], [11], [12]. Although *Candida albicans* accounts for the majority of infections, other non-albicans *Candida* species such as *Candida tropicalis* have increasingly been recognized as pathogens for these types of infections. *C. tropicalis* is considered the leading pathogen in nosocomial fungemia and hepatosplenic fungal infections in patients with cancer, especially leukemia [13], [14]. *C. tropicalis* is the second most frequently isolated non-albicans pathogen in the Asia-Pacific region and in Brazil [15], [16]. In large independent epidemiologic surveys, the isolation rate of *C. tropicalis* from blood is 5% to 30% [17], [18]. In evolutionary terms, it is closely related to *C. albicans* [19]–[21]. Previous studies conducted in Asia show the frequency of fluconazole resistance in the intermediate *C. tropicalis* strains, which was originally found in *Candida glabrata* that displayed natural resistance to fluconazole [22], [23]. Furthermore, a high proportion of *C. tropicalis* isolates has exhibited low susceptibility to flucytosine [24], [25].

Numerous molecular typing methods have been used to determine the molecular epidemiology and resistance of *C. tropicalis*, such as MLST (multilocus sequence typing). MLST is a useful tool in population analysis within a species [20], [26]. The MLST data is comparable among labs worldwide via the central database in the Internet (http://www.mlst.net/), and it satisfies the increasing need for global surveillance [27]. The MLST approach reveals different geographical origins, anatomic sources, and other characteristics between clades of closely related isolates [20]. MLST has been developed for *C. albicans* and *C. glabrata* [28]–[30], which offers further information regarding strain variations, including maintenance, replacement, and microevolution in the host [27]. The MLST system for *C. tropicalis*, which comprises six housekeeping genes (*ICL1*, *MDR1*, *SAPT2*, *SAPT4*, *XYR1* and *ZWF1a*), has been described by Tavanti et al. in 2005 [24].

The aim of our study is to examine the clonality of *C. tropicalis* by performing successive strain collection for one year in a general hospital in Beijing, China, and to ascertain whether factors such as hospital department origin, anatomic source, and so on are related to certain specific MLST diploid sequence types (DSTs) types. Furthermore, the correlation between the 52 DSTs of the isolates and the 268 DSTs that represent the international strains in the central database was analyzed through a minimum spanning tree (MST) using the BioNumerics software.

## Methods

### *Candida tropicalis* isolates

All of the isolates included in this study were collected from the clinical laboratory of a general hospital in Beijing, China during a period of 1 year (between August 2010 and September 2011). A total of 58 *C. tropicalis* strains were identified from the departments of the hospital (Table 1). These isolates were obtained from different specimens, namely, sputum/throat swab, urine, feces, vaginal/prostatic secretions, drainage/sanies, and blood. All isolates were identified by ITS sequencing and AUX 20C (bioMérieux) in our lab. The universal primers ITS1 and ITS4 were used to amplify the ITS fragment and to sequence it at both directions [31]. All 58 isolates were collected from hospitalized patients except 5, which were from the Outpatient Department. Detailed information regarding these isolates is summarized in Table 1. The strains were stored at −80°C in brain–heart infusion (Oxoid). The isolates were maintained on Sabouraud agar (Oxoid) during the study.

**Table 1:** *C.tropicalis* isolates, isolate sources and genotypes determined by MLST analysis.

Isolate	Anatomic source	Isolation Date (y/m)	Dept in hospital	DST	MLST type
BZR-2	Sputum	2010/08	Geriatrics	269	sigleton
BZR-3	Prostatic secretion	2010/08	Thoracic surgery	270	sigleton
BZR-4	Sputum	2010/08	Emergency Dept	271	sigleton
BZR-7	Sputum	2010/08	EICU	272	group6
BZR-14	Urea	2010/09	Immunology Dept	273	group2
BZR-20	Feces	2010/09	Pediatrics	274	group2
BZR-22	Urea	2010/09	EICU	275	group3
BZR-25	Sputum	2010/09	Emergency Dept	276	group5
BZR-31	Sputum	2010/09	Respiratory Dept	277	group5
BZR-36	Throat swab	2010/10	Immunology Dept	278	group2
BZR-38	Blood	2010/10	ICU	279	group1
BZR-39	Sputum	2010/10	Respiratory Dept	280	sigleton
BZR-41	Urea	2010/10	ICU	278	group2
BZR-51	Sputum	2010/11	Out-patient Dept	281	sigleton
BZR-52	Urea	2010/11	EICU	282	sigleton
BZR-53	Drainage	2010/11	Respiratory Dept	283	sigleton
BZR-54	Feces	2010/11	Hematology specialty	284	sigleton
BZR-56	Sanies	2010/11	Respiratory Dept	285	sigleton
BZR-61	Sputum	2010/11	Immunology Dept	286	group6
BZR-62	Urea	2010/12	EICU	287	group4
BZR-63	Vaginal secretion	2010/12	Out-patient Dept	288	sigleton
BZR-64	Sputum	2010/12	Respiratory Dept	289	sigleton
BZR-66	Urea	2011/01	Geriatrics	290	sigleton
BZR-67	Feces	2011/01	ICU	279	group1
BZR-68	Sputum	2011/01	Respiratory Dept	291	group3
BZR-69	Vaginal secretion	2011/01	Out-patient Dept	292	group6
BZR-70	Sputum	2011/01	Emergency Dept	293	group4
BZR-71	Sputum	2011/02	Respiratory Dept	294	group4
BZR-73	Sputum	2011/02	Respiratory Dept	295	group6
BZR-74	Sputum	2011/02	ICU	296	group2
BZR-75	Sputum	2011/02	Respiratory Dept	297	sigleton
BZR-76	Vaginal secretion	2011/02	Out-patient Dept	298	sigleton
BZR-78	Sputum	2011/03	Hematology specialty	277	group5
BZR-79	Sputum	2011/03	Hematology Dept	299	sigleton
BZR-80	Sputum	2011/03	Geriatrics	300	sigleton
BZR-81	Sputum	2011/04	Emergency Dept	301	group2
BZR-82	Sputum	2011/04	Respiratory Dept	302	sigleton
BZR-84	Feces	2011/04	Gastroenterology Dept	279	group1
BZR-85	Sputum	2011/04	Respiratory Dept	303	group1
BZR-87	Throat swab	2011/05	EICU	304	sigleton
BZR-88	Sputum	2011/05	Hematology Dept	305	sigleton
BZR-89	Sputum	2011/05	Geriatrics	306	sigleton
BZR-90	Sputum	2011/05	Respiratory Dept	279	group1
BZR-92	Urea	2011/05	Respiratory Dept	307	sigleton
BZR-95	Sputum	2011/06	Geriatrics	308	group1
BZR-97	Feces	2011/06	General Surgery	309	group5
BZR-98	Urea	2011/06	Geriatrics	310	sigleton
BZR-99	Sputum	2011/07	ICU	311	group3
BZR-100	Urea	2011/07	Out-patient Dept	312	group6
BZR-103	Urea	2011/07	Geriatrics	279	group1
BZR-104	Sputum	2011/07	Immunology Dept	313	group2
BZR-105	Vaginal secretion	2011/08	ICU	314	sigleton
BZR-106	Sputum	2011/08	Geriatrics	315	sigleton
BZR-115	Drainage	2011/08	General Surgery	316	sigleton
BZR-116	Drainage	2011/09	ICU	317	group3
BZR-117	Sputum	2011/09	ICU	318	sigleton
BZR-119	Sputum	2011/09	EICU	319	group6
BZR-120	Sputum	2011/09	EICU	320	sigleton

### DNA extraction, PCR amplification and sequencing of *C. tropicalis* strains

The total genomic DNA of the isolates was extracted using a Yeast DNA Purification Kit (Tiangen) according to the manufacturer's instructions. The DNA concentrations were estimated with a spectrophotometer absorbance at 260. DNA extracts were stored at −20°C until use. Amplification were carried out in a final reaction volume of 50 µl that consisted of 25 µl of Premix rTaq (TAKARA), 20 µl of dH_2_O, 3 µl of template DNA, and 1 µl of the forward/reverse primers. The six multilocus loci of *C. tropicalis* were *ICL1*, *MDR1*, *SAPT2*, *SAPT4*, *XYR1* and *ZWF1a*, as previously described [24]. The primers and the detailed amplification conditions for these fragments were in accordance with methods described in previous studies [24], [32]. The amplified fragments were purified using a PCR purification kit (Qiagen) according to the manufacturer's instructions. Both strands of the purified fragments were sequenced using the same primers as those used in the initial amplification. DNA sequencing was performed with an ABI 3730 DNA Analyzer (Applied Biosystems).

### MLST analysis of *C. tropicalis* strains

The MLST data were based on six genes, namely, *ICL1*, *MDR1*, *SAPT2*, *SAPT4*, *XYR1*, and *ZWF1a* [24]. Sequences of both strands were aligned using the DNASTAR software (http://www.dnastar.com). The allelic profiles and the DSTs of the six gene sequences were obtained from the *C. tropicalis* MLST sequence-type database (http://pubmlst.org/ctropicalis/). The new allelic profiles found in this study were submitted to the curator of the *C. tropicalis* MLST database and were assigned new allele numbers (Table 1). Phylogenetic analysis of the isolates were determined through cluster analysis using UPGMA (unweighted pair group method using their arithmetic averages) of the BioNumerics software version 5.1 (Applied Maths, Kortrijk, Belgium) based on the variable sites from the six gene fragments conjoined into a single sequence as described by Tavanti et al. [24], [33]. The MLST groups were determined using the sequence similarity threshold of the UPGMA dendrogram, which was based on the statistical significance of the cluster nodes from bootstrapping with 1000 replications. The ratio of non-synonymous to synonymous sequences of the six gene fragments was calculated using the SAPT2 software in the MLST website (http://pubmlst.org/software/analysis/). The MLST clonal clusters were determined using the eBURST package (http://eburst.mlst.net/). The eBURST algorithm places all related isolates into clonal complexes and, whenever possible, predicts the founding or ancestral DST of each complex. The genetic relationship between the 58 isolates in our study and the 405 isolates in the central data library were further analyzed using the minimum spanning tree (MST) of the BioNumerics software. This method determines the putative relationships between isolates and records isolates as related when one of the six MLST gene loci are different. Discriminatory power was calculated by the application of the Simpson index [34].

## Results

### *C. tropicalis* strain isolated and its differentiation by MLST

**Figure 1:**
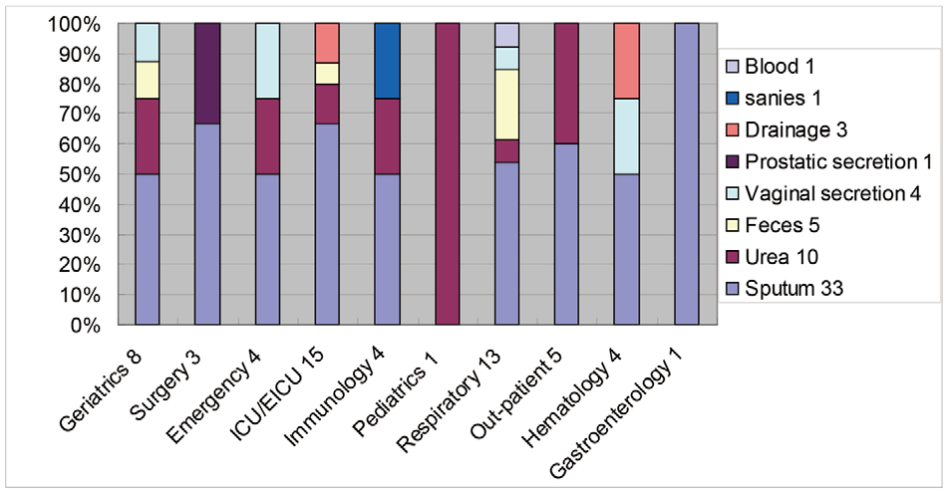
Distribution of the *C. tropicalis* isolates according to separate source/medical dept. The number following the dept name is the total strains isolated from the according dept. ICU, Intensive Care Unit; EICU, Enhanced Intensive Care Unit; Dept, Department. The number following the word is No. of *C. tropicalis* strains isolated.

A total of 58 *C. tropicalis* strains were identified during the study period (August 2010 and September 2011) (Table 1). Each isolate was from a unique patient. The distribution of the isolated *C. tropicalis* strains is shown in Figure 1. All strains were susceptible to flucytosine, amphotericin B, fluconazole, itraconazole, and voriconazole (data not shown).

All 58 isolates were subjected to MLST analysis to determine their genetic relationship. A total of 2,677 bp from the six gene loci (*ICL1*, *MDR1*, *SAPT2*, *SAPT4*, *XYR1*, and *ZWF1*a) were sequenced in each of the 58 isolates and 28 (1.05%) polymorphic nucleotide sites were found. Up to 10 new alleles were found in the fragments of *ICL1* (2), *MDR1* (3), *SAPT4* (1), *XYR1* (2), and *ZWF1*a (2), which accordingly generated new genotypes. Up to 52 DSTs were differentiated among the 58 unique isolates based on the six sequenced fragments (Table 1). All 52 of the DSTs of strains from Beijing, China were not found in the internet DST database, and were added to the central *C. tropicalis* MLST database. The *SAPT4* locus contained 10 polymorphic amino acids, the *MDR1* locus has 6, the *ICL1* and *SAPT2* loci has 2, and the *XYR1* and *ZWF1*a loci has 1 (Table 2). The percentage of polymorphic sites per gene was as follows: 0.45% (*ICL1*), 1.41% (*MDR1*), 0.38% (*SAPT2*), 2.56% (*SAPT4*), 0.27% (*XYR1*), and 0.19% (*ZWF1*a). Among the six sequenced gene loci, *XYR1* was the most specific, distinguishing 15 genotypes per polymorphism for only 1 polymorphic site (Table 2), whereas *SAPT4* was the least efficient gene (Table 2). Most isolates were discriminated based on a smaller number of polymorphisms in each fragment than those listed in Table 2. Large numbers of polymorphic sites were found in *SAPT4* and *MDR1*, especially in strains BZR 67, BZR 84, BZR 85, BZR 90, BZR 95, BZR 103, and BZR 105, which indicates the heterozygosity or the homozygous of these sites. Depending on the sequence polymorphisms of all fragments, the ratios of the non-synonymous to the synonymous amino acid changes were less than 1, which suggests that the genes were under neutralizing selective pressure (Table 2).

**Table 2:** Properties of 6 gene fragments of *C. tropicalis* used for MLST of 58 isolates.

Gene Loci	No. of polymorphic sites	No. of genotypes	No. of genotypes/polymorphism	Polymorphic amino acids	Ratio of Nonsynonymous to synonymous changes
*ICL1*	3	7	2.33	2	0.0393
*MDR1*	6	15	2.5	6	0.0092
*SAPT2*	2	5	2.5	2	0.0212
*SAPT4*	13	18	1.39	10	0.1075
*XYR1*	1	15	15	1	0.0357
*ZWF1a*	3	10	3.33	1	0.0277

### Population structure of 58 isolates from separate sources

**Figure 2:**
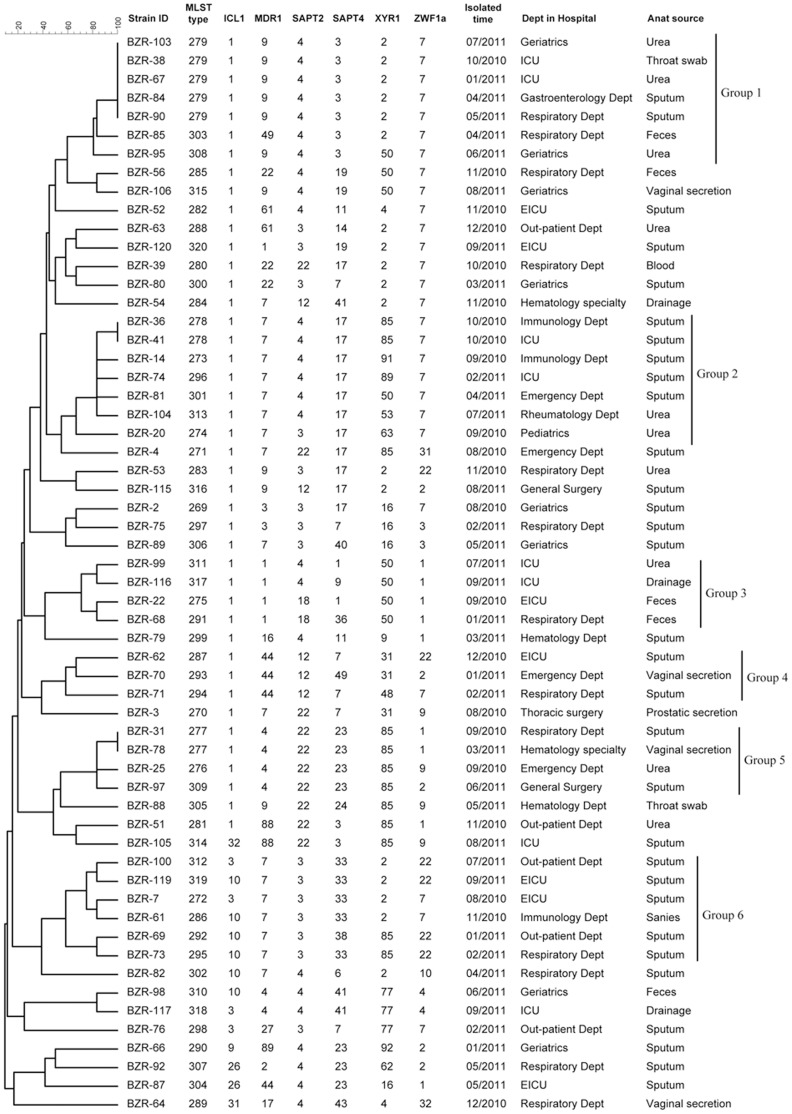
UPGMA dendrogram indicating similarities 58 *C. tropicalis* isolates determined by MLST of six gene loci. Groups were defined by a sequence similarity of >80%.

The dendrogram in Figure 2 indicates the similarities of 58 *C. tropicalis* isolates, as determined using the MLST analysis of the six gene fragments. No large group was generated from the 58 isolates based on the UPGMA dendrogram. Most of the isolates were dispersed and a few clustered as small groups (Figure 2). The dendrogram shows that 6 groups (1–6) were generated based on the bootstrap value, and the majority of isolates (32 in 58) appeared as unrelated singletons (Figure 2). Group 1 had 4 DSTs, group 2 had 6, group 3 had 4, group 4 had 3, group 5 had 3, and group 6 had 6. Except for BZR 103, BZR 38, BZR 67, BZR 84, BZR 90, BZR 36, BZR 41, BZR 31, and BZR 78, all isolates from the same patient but from different specimens or different isolation times displayed different MLST types. No obvious relationship was found between DST and source specimens or medical department. In group 1, five strains had the same genotype, DST 279; three were isolated from different medical departments and two were from the ICU. Strains BZR 36 and BZR 41 from different departments had the same DST, DST 278, and they were classified into group 2. DST 277, composed of BZR 31 and BZR 78, were from the distinct source specimens of two medical departments. No relationship was observed between the individual isolates and anti-fungal resistance. Up to six clonal clusters were generated in eBURST, which corresponded to the six UPGMA clusters except for group 4. The rest of the isolates were dispersed as singletons, similar to the UPGMA dendrogram. The two eBURST clonal clusters (DSTs 278, 296, 273, 301, and 313; DSTs 272, 286, 319, 312, 295, and 292) had the predicted founding types 301 and 319 and are related to UPGMA groups 2 and 6, respectively. The remaining eBURST clonal clusters had no predicted founding type. All but one of the isolates in the group 1 was in the eBURST clonal cluster (DSTs 285, 315, 308, 279 and 303), which is also related to group 1. The other two UPGMA clusters, groups 3 and 5, were also correlated with eBURST data. No genotypes were correlated with specimen type, hospital origin, and fluconazole resistance.

### Comparison of 52 DSTs to 268 DSTs in *C. tropicalis* database

**Figure 3:**
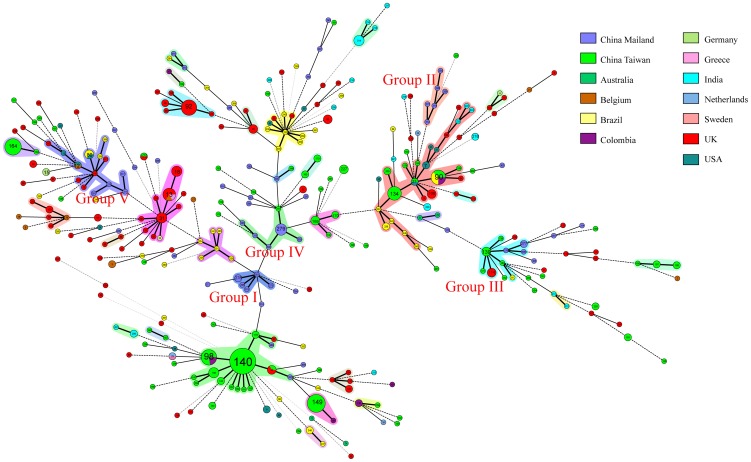
Minimum spanning tree of the 58 *C. tropicalis* isolates (52 DSTs) and the 401 isolates (268 DSTs) in the central database. ICU, Intensive Care Unit; EICU, Enhanced Intensive Care Unit; Dept, Department.

The 52 DSTs were compared to all 268 DSTs that represent the international strains in the MLST website through a minimum spanning tree (MST) using BioNumerics software. In the MST dendrogram, more than half of the 52 DST types (30) assembled as a clonal cluster (Figure 3). Five relatively large clonal clusters were generated from the isolates from Mainland China, in which three complexes (I, II, and IV) were clustered with the isolates from Mainland China except for the isolate (DST 177) from Taiwan, which was included in complex IV (Figure 3). In the other two cluster complexes (III and V), isolates from China Mainland were primarily co- clustered with isolates from Taiwan, Europe or USA (Figure 3). The remaining isolates from Mainland China were dispersed as singletons (Figure 3). In clonal complex III, five DSTs in the present study (276, 277, 359, and 305) clustered with strains from Taiwan, three from Europe, and one from Brazil. Four DSTs (291, 275, 317, and 311) clustered with six from Belgium, five from Brazil, and three from USA to form cluster complex V. In a previous study, Tavanti et al. found six flucytosine-resistant *C. tropicalis* isolates, two of which were scattered as singleton and the rest formed two clusters in the MST dendrogram (Figure 3) [24]. No DST type from the isolates from Mainland China clustered with the flucytosine-resistant isolates, which indicates that those isolates were susceptible to antifungal agents in the vitro test. For the fluconazole-resistant isolates from Taiwan reported by Chou et al. in 2007 [22], a major cluster with DST 140 formed the core type in the MST dendrogram (Figure 3). Two DST types (DSTs 269 and 297) susceptible to fluconazole in vitro test -clustered with the clonal cluster from Taiwan (Figure 3). The isolates from Mainland China, Taiwan, Belgium, Brazil, the US, and India generated relatively independent clonal clusters (Figure 3), which indicates the geographic evolution of *C. tropicalis*. In the cluster with DST 140 as the main type, only DST 269 and 297 were included (Figure 3), whereas the cluster complex composed of isolates from Belgium and Brazil included few of the isolates from Mainland China (Fig. 3). In cluster complex III, the isolates from Mainland China with DST types 276, 277, and 309 clustered with isolates from Taiwan (DSTs 138, 139, 171, and 184), Belgium (DSTs 114 and 128), and Brazil (DST 91). Meanwhile, in complex V, DSTs 291, 275, 317, and 311 from the isolates from Mainland China clustered with isolates from Belgium (DSTs 50, 12, 111, 110, 119, and 78), Brazil (DSTs 80, 249, 256, and 266), and USA (DST 1, 12, and 80). This may indicate that *C. tropicalis* strains with these DST types may be closely related, and are relatively conserved and differentiated regardless of geographic location.

## Discussion

*C. tropicalis* is considered as the leading cause of nosocomial fungemia and hepatosplenic fungal infections in patients with hematologic malignancies [13], [14]. In this study, most *C. tropicalis* strains were isolated from sputum (33) and urine (10) specimens. Although the positive sputum specimens were identified using three-time continual culture, classifying the isolation of *Candida* species from the upper respiratory tract as colonization or infection is still debated [35]. Isolation of *C. tropicalis* from urine and blood, which are ideally abacterial body fluids, is strong evidence of candidemia and candiduria. Only one blood infection was identified in the present study, much lower than that in a similar study [1], [8], [17], which suggests that the candidemia caused by *C. tropicalis* may vary among independent hospitals.

The six gene fragments chosen yielded more than one genotype per polymorphic locus (Table 2). The number of polymorphic sites in the present study is relatively lower than that in a previous study [22], [24], [33]. However, the number of one genotypes per polymorphic locus is still much higher than that previously reported (Table 2) [22], [24], [33], which may indicate the high diversity of *C. tropicalis* strains in Mainland China. *XYR1* showed the highest typing efficiency in the present study and in the study by Tavanti et al. in the UK [24]. The number of genotypes per polymorphic locus in the present study is three times higher than that in the study by Tavanti. However, Tavanti reported considerably fewer polymorphic sites than in the present study. This discrepancy may due to the difference in the number of *C. tropicalis* isolates or it may indicate the stronger discriminating power of *XYR1* gene among the *C. tropicalis* strains from Mainland China. This result may also be because some of the isolates analyzed before were from the same patients unlike the 58 isolates in the current study, which were from different patients. *SAPT4* was the least efficient fragment in terms of the number of genotypes per polymorphic locus in the present study and that conducted by Chou et al. [22], which indicates that the discriminatory power of the six gene fragments may vary according to country of location and the number of isolates.

A total of 52 DSTs and 70 genotypes were found (Table 1). All 52 DSTs were novel to the *C. tropicalis* MLST central database. Isolates with the same DST (BZR 103, BZR 38, BZR 67, BZR 84, and BZR 90; BZR 36 and BZR 41; BZR 31 and BZR 78) were from the same specimen sources and hospital departments (Figure 2). In this study, the 58 isolates that included 8 cluster complexes (30 strains), distributed in 8/32 of the global MST clusters (Figure 3). The remaining 28 isolates were distributed independently in the MST global tree. In the MST tree, most of the isolates from Mainland China, Taiwan, Belgium, Brazil, and USA formed independent clusters, which indicates that *C. tropicalis* may evolve according to different locations. DST 277, 278 and 279 shared over 3 strains were relative the dominating genotype formed in Mainland China. While in Taiwan, several major genotypes were generated, such as DST90 (shared by Brazil and Colombia strains), DST 98 (shared by Taiwan and Colombia), DST134, DST 140 (a main genotype of a clonal cluster showing resistance or trailing growth of fluconazole), DST 149 and DST 164 [22]. In rest countries, only a few genotypes were produced in UK (DST 92, 31 and 13 shared Belgium and Greece) and in Indian (DST 214). Weather those genotypes (DST 80, 12, 45, 23 and 27 et al.) shared by more than two countries suggests more widespread ability and potential epidemic need further study. No genotypes produced in Mainland China was shared by other counties, which may give a cue that the epidemiology of *C. tropicalis* in China is relatively independent from other area. More collection of strains and molecular typing data are needed for better understanding the epidemic character of *C. tropicalis* in China, and finding potential outbreak genotypes in future. Similar to *C. albicans*, *C. tropicalis* strains from one patient are generally highly similar whereas those from different patients differ considerably [20], [36]. In this study, genotype of each *C. tropicalis* isolate was patient-specific and unrelated to type specimen of the isolate, the hospital department, or the fluconazole resistance pattern. For *C. albicans*, no significant association has been observed between the MLST type of each isolate and fluconazole resistance [32]. However, in 2007, Chou et al. reported that the DNA types of *C. tropicalis* are related to fluconazole resistance [22]. Furthermore, two independent study groups, one in the UK and one from France, found flucytosine-resistant clonal complexes in 2005 and in 2008, respectively [24], [25]. The *C. tropicalis* from Mainland China in the current study were all susceptible to fluconazole and flucytosine (data not shown), which indicates that the antifungal resistance may develop geographically. It could be inferred that the population structure of *C. tropicalis* from Mainland China is similar with it from other countries (no obvious clonal cluster generated correlated with specimen source, patients age or hospital origin), except for fluconazole/flucytosine resistance.

We have added the information on *C. tropicalis* to the growing list in the MLST central database and our study may be useful for further studies on the similarity, genetic relationship, and molecular epidemiology of *C. tropicalis* worldwide. The expansion of the *C. tropicalis* MLST database (http://pubmlst.org/ctropicalis/) will reveal the relationship of the population structure of *C. tropicalis* strains with the clinically relevant properties of clades and geographic locations. And further study of the population structure will benefit the epidemiological survey of *C. tropicali* in China. Our data could be compared with MLST data from outbreak strains in epidemic hospitals in China, which is helpful for tracking the origin of the outbreak.
